# A Vision-Based Approach for Sidewalk and Walkway Trip Hazards Assessment

**DOI:** 10.3390/ijerph17228438

**Published:** 2020-11-14

**Authors:** Rachel Cohen, Geoff Fernie, Atena Roshan Fekr

**Affiliations:** 1 The Kite Research Institute, Toronto Rehabilitation Institute—University Health Network, University of Toronto, Toronto, ON M5G A2A, Canada; Geoff.Fernie@uhn.ca (G.F.); Atena.RoshanFekr@uhn.ca (A.R.F.); 2 Institute of Biomedical Engineering, University of Toronto, Toronto, ON M5S 3G9, Canada

**Keywords:** trips and stumbles, falls, sidewalk, tripping hazards, edge detection, error prediction, calibration

## Abstract

Tripping hazards on the sidewalk cause many falls annually, and the inspection and repair of these hazards cost cities millions of dollars. Currently, there is not an efficient and cost-effective method to monitor the sidewalk to identify any possible tripping hazards. In this paper, a new portable device is proposed using an Intel RealSense D415 RGB-D camera to monitor the sidewalks, detect the hazards, and extract relevant features of the hazards. This paper first analyzes the effects of environmental factors contributing to the device’s error and compares different regression techniques to calibrate the camera. The Gaussian Process Regression models yielded the most accurate predictions with less than 0.09 mm Mean Absolute Errors (MAEs). In the second phase, a novel segmentation algorithm is proposed that combines the edge detection and region-growing techniques to detect the true tripping hazards. Different examples are provided to visualize the output results of the proposed method.

## 1. Introduction

Tripping is a major concern especially for elderly people, as it is a primary cause of falls leading to serious injuries. Cracked pavement, loose tiles, bumps, and potholes are just a few examples of sidewalk hazards that cause trip-related injuries and remain undetected. Cities in Ontario, Canada are liable for damages to a person if they do not repair raised edges in sidewalks greater than 20 mm (0.75 in) on public property according to the Minimum Maintenance Standards [1]. The International Building Code in the United States calls for beveling any changes in elevation greater than 6 mm (0.25 in) [2]. Cities only perform sidewalk inspection once a year, as the inspection alone costs the city thousands of dollars [1]. For example, in the city of Hamilton, the sidewalk inspections cost about $60,000 annually, and still the city’s Risk Management department is concerned about the accuracy of inspection information [3]. In 2012, Hamilton paid $2.5 million in liability claims due to stumbles over cracks in sidewalks [4]. The inspection is often done manually by summer students, patrolling the city’s sidewalks and measuring the depths and size of the cracks with a ruler. This method is not only unable to provide accurate and consistent measurements, but it is also time consuming and expensive.

To the best of our knowledge, no systematic way exists to monitor and scan sidewalks. Walkway hazards are particularly dangerous, since they are often difficult to see. The risk of not seeing the hazards can be increased when people are distracted by diverse reasons such as texting, talking on the phone, holding a child’s hand, or by simply looking at the merchandise in a store. In addition, the risk may increase when there is not enough lighting at night, making the danger even more difficult to perceive. Automated sidewalk inspection is desirable for the city and government, as it provides an accurate assessment of the hazards while being economical. Automatic inspection will prevent lawsuits, eliminate the need for manual inspection, and prevent injuries.

In this paper, a novel cost-effective portable sidewalk monitoring system is proposed that scans the sidewalk and outputs a 3D image of the hazard to extract the important features such as average depth, maximum and minimum depths, and the size of the hazard. We first evaluate different Machine Learning (ML) models to predict the error of the chosen device, the Intel RealSense D415, considering different environmental factors. Once the error is predicted, it can be eliminated to obtain a more accurate depth image, which is crucial for the detection of cracks. Subsequently, we proposed a novel algorithm to detect tripping hazards using a combination of region-growing and edge segmentation with the depth map output from the camera. The combination of these two goals creates a device that accurately scans sidewalks to determine tripping hazards.

## 2. Literature Review

### 2.1. Pavement and Crack Analysis

Several attempts have been conducted to scan pavement with various technologies [5,6,7,8,9,10,11,12,13,14,15,16,17,18,19,20]. Ai et al. used Light Detection and Ranging (LiDAR) technology to detect characteristics in sidewalks including width, grade, and slopes [5]. LiDAR instruments have been used to extract the landscape characteristics [21,22,23,24], however based on a study in the US [24], the accuracy of LiDAR is typically 15–20 cm (~6 in) and 1/3–1 m for vertical and horizontal measurements, respectively. Therefore, this technology does not provide enough accuracy to detect small cracks and their depths. Research has also been conducted to detect tripping hazards on construction sites using a variety of depth and color camera fusion approaches [6]. These methods were mainly used to detect large objects using the Microsoft Kinect V2 [6]. Bitelli et al. used laser scanning techniques on various types of asphalt to characterize the texture and road performance [7]. This method used triangulation lasers to determine the pavement’s quality and roughness for the evaluation of the safety of the road [7]. Although an accurate surface profile was achieved, it was not used to detect cracks and was mainly used for classifying pavement types [7]. A commercially available, patented device called the Fugro Roadware Automatic Road Analyzer (ARAN), used in the United States, is attached to motor vehicles that survey highways at a high speed to monitor road conditions and detect cracks in the asphalt [8]. A light illuminates the pavement as a vehicle passes, and stereoscopy is used to measure the depths [8]. Other devices are the RoadCrack used in Australia, the PAVUE used in the Netherlands, and HARRIS used by the UK Transport Research Lab (TRL) [25]. A study by the TRL shows that these commercial systems have difficulty when detecting joints, patches, road markings, and road edges, leading to over detection [25]. In addition, these devices are not applicable to sidewalks. Other studies used RGB cameras with image processing, morphological operations, or machine learning to detect the cracks in concrete [9,10,11,12,13,14,15,16,17,18,19]. Zhang et al. used Convolutional Neural Networks on still photos to determine if a crack was present [20]. They found that the precision, recall, and F-score were superior with the deep learning algorithm compared to Support Vector Machine or Boosting algorithms [20]. However, these devices do not provide any information regarding the depth and size of the cracks, as they only used RGB images.

### 2.2. Comparison of Available Depth Measurement Techniques and Devices

Depth measurement technologies have been developed for a variety of applications [26,27,28,29,30,31,32]. The accuracies of these techniques can vary from sub-millimeter to centimeters. The laser triangulation method uses trigonometry to calculate the depth of the object. A single laser beam is projected onto the object, and the reflected beam falls incident on the receiving sensor at a certain angle [26]. This angle is used to calculate the distance [26]. Laser triangulation is an accurate method and works best on flat, non-reflective surfaces [27]. It can output depth with an accuracy and precision in the micrometer range. However, this technology is prone to error when measuring textured and uneven surfaces, as in the case of sidewalks. In this case, the laser can be deflected and not transmitted back to the receiving sensor [28]. Additionally, many of these devices output a single depth value to their built-in display, which adds a step of transferring the data to a computer, making it difficult to post process the data to improve the accuracy.

Unlike triangulation, structured light and stereoscopy technology provide a depth map over a grid of pixels instead of the depth of a single point, which is more appropriate for our application. Stereoscopy is a method of depth detection that uses bipolar vision similar to the human eyes. Two cameras are used to capture the scene, with a known offset between the two, which is called disparity [29]. The two images are combined to give the depth of each pixel in the scene. Stereoscopy works best in high exposure and cannot capture smooth surfaces easily [30]. To combat this, the Intel RealSense Depth Camera D400-series can perform active stereoscopy, where an array of light beams is projected onto the surface in an attempt to mimic a textured surface [31]. This method of active stereoscopy is similar to structured light methods. Structured light cameras create a depth map by emitting different patterns or structures of laser points onto the scene. As the array hits the surface, it is deformed, and a camera calculates the depth based on the level of deformation [30]. There are several devices that use this technology for 3D scanning applications, including the Orbecc Astra-S and the Intel RealSense SR300. Structured light does not provide accurate measurements in outdoor environments, since it is sensitive to optical interference, such as sunlight [30].

A study by Giancola et al. showed that the Astra-S had a larger bias at 1000 mm compared to the Kinect V2 and the depth camera D415, despite having a lower uncertainty [30]. It was also more sensitive to ambient light changes in the outdoor environment and performed better on non-textured surfaces [30]. Vit and Shani’s study compared the Astra-S, the Intel SR300, Intel D435, and the Kinect V2 for object detection under various natural lighting conditions [33]. For all lighting conditions, the Intel D435 outperformed the others by providing higher quality depth information with a higher fill rate and more accurate size estimation [33]. The Astra-S underperformed in nearly all categories, and the SR300 was applicable only for short ranges, as expected [33]. Carfagni et al. compared the D415 to its predecessor the SR300 and found that they performed relatively similarly at short ranges [34]. Intel has stated that the D415 depth camera performs better than the D435 for short-range and high-accuracy applications [31].

Several devices and methods were investigated for our application. The main criteria were to be cost effective and to be able to detect depth accurately in lit areas on textured surfaces. Triangulation was investigated as it is accurate but only measures single points at a time, and it does not work well on textured surfaces [27]. An array of 20 Time of Flight (ToF) sensors was also tested but did not provide accurate enough results for our application. Considering the price, availability, depth reading range, and accuracy results found in [30,33,34], the D415 depth camera was chosen for extracting the features of tripping hazards in different environmental conditions. This is an economical solution as the RealSense D415 only costs $149 USD [35].

### 2.3. Current Calibration Techniques for Depth Cameras

Calibration is an important step to achieve a highly accurate representation of the real world in the captured images. Calibration is used to determine the relation between the camera’s pixels and the real world units (e.g., millimeters). Generally, even after calibration, the errors may not be fully removed. The RealSense cameras are factory calibrated, and it is recommended not to re-calibrate the device unless there are significant errors including a subpixel error above 0.2, poor fill rate, or an error greater than 3% [36,37]. The dynamic calibration performed on the RealSense device is twofold: (1) A rectification calibration is done to reduce the holes in the depth image and increase fill rate, (2) A depth scale calibration is done to align the depth and camera images [36]. The dynamic calibration optimizes the extrinsic parameters, and the intrinsic parameters such as distortion, principal point, and field of view are not dynamically calibrated. According to Intel’s documentation [36], after factory calibration, there is still a possibility of having up to 3% error, which will have a large impact on our specific application on the depth measurement of tripping hazards. In general, the RealSense documentation indicates that the error is on average 2% at a 2 m distance and decreases as the device is moved closer to the scanned object.

Various studies proposed calibration procedures and techniques for RGB-D cameras. Zhang et al. created one of the most widely used camera calibration techniques, using a checkerboard pattern with a known size [38]. There have been several improvements to this technique over the years. Fuersattel et al. developed a novel way to perform stereo calibration to align laser scanner and RGB information [39]. The maximum accuracy obtained was 1.3 mm or 0.2 pixels [39]. Cabrera et al. attempted to remove the RMS (Root Mean Square) error. This method could be used on all types of RGB-D cameras (structured light, time of flight, or stereo) by using a common checkerboard technique [40]. A curve-fitting technique was used to obtain the RMS as a function of distance to the checkerboard and was tested using Kinect and Zed devices [40]. Chen et al. also created a calibration technique that shows the error as a function of distance to the scanned object, using motion capture [41]. Yamazoe et al. developed a calibration technique for the Kinect based on Zhang’s method with a novel way to estimate intrinsic parameters [42]. These calibration techniques only consider the error versus distance to the scanned object or errors within the camera’s intrinsic properties and do not consider other factors including environmental parameters such as temperature and light. Therefore, this paper attempts to find an adaptive calibration technique that considers different factors such as light, temperature, distance from the object, the angle of the camera, and the pixel depth, as well as the camera’s different resolutions. This technique will enhance our ability to extract the tripping hazards features accurately.

### 2.4. Region Growing and Edge Detection

Image segmentation is a fundamental task in crack detection. Region-based and edge-based segmentation are two of the main image segmentation classifications. The process of region growing, introduced by Adams and Bischof, takes one seed (a starting pixel) and finds neighboring pixels using a threshold to categorize the pixels into regions [43]. Since then, several improvements have been made to the region-growing algorithms, mainly in the seed selection procedure [44,45,46,47,48,49,50,51]. For example, Isa et al. used k-mean clustering to modify seed selection of the edge detection algorithm analyzed on Pap smear images [44]. Fan et al. developed a method to automatically select a seed by taking the centroid of the edge regions as the initial seed [45]. Different studies showed that the fusion of edge and region information can improve the segmentation algorithms [44,47,48,50,51]. Wang and Yang used edge linking and region grouping to segment images by detecting and linking the edges to form contained regions [46]. Pavlidis and Liow found that combining region growing and edge-based methods can provide better results compared to using each method individually [47]. Luo et al. tested a new algorithm on ultrasound images that used a multi-objective particle swarm optimization to combine edge and region information to segment the image [48]. Chen et al. used a Canny edge detection output as one of the seed selection conditions to achieve initial seeds [49]. In this paper, we used a similar approach; however, we took all edges detected by the Canny method as seeds to effectively grow regions around the edges based on a customized threshold value. This threshold was obtained from the standards for sidewalk tripping hazards [2] and can be easily changed if required.

## 3. Materials and Methods

The Intel RealSense depth camera D415 was used which utilizes stereoscopy with an Active IR Stereo and an integrated RGB sensor. This camera has a Field of View (FOV) of 65° in the horizontal direction, 40° in the vertical direction, and 72° in the diagonal direction. The resolution ranges from 424 × 280 to 280 × 720. The frame rate ranges from 6 to 90 fps with a rolling shutter on the depth sensor.

In this paper, a new method is proposed to detect sidewalk cracks by the Intel RealSense D415 using a combination of region and edge segmentation. Several ML models were evaluated to predict the errors and correct the pixel values, accordingly. Different feature selections are used to see which factors contribute the most to our prediction error. The output from the best ML model was removed from the pixels to obtain the final calibrated images. Subsequently, image processing algorithms were performed on the calibrated images to estimate the characteristics of the tripping hazards.

### 3.1. Error Elimination Algorithms

#### 3.1.1. Data Collection

**Figure 1:**
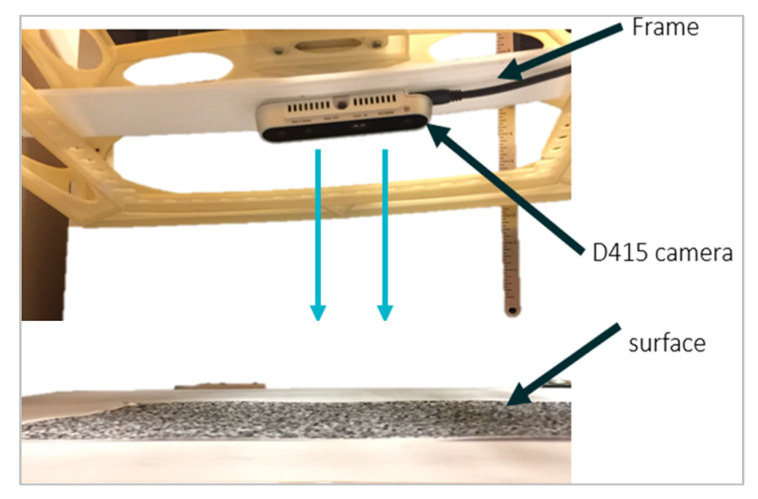
Test rig for D415.

The camera was attached to a leveled test rig and pointed downward at the surface of interest to capture data, as shown in Figure 1. Various data collections were performed to analyze the environmental effects on the camera.

For training our ML model, the data were collected on leveled rough surfaces (textured but with no large cracks or bumps) in both indoor and outdoor environments. The indoor trials were performed on either flat carpet or on a flat wooden surface with a printed image of a rough surface placed on top. The outdoor data were collected on flat concrete or asphalt with no cracks. The entire surface was scanned to obtain each pixel’s depth reading, and the center region was selected as the Region of Interest (ROI). The disparity chosen for each trial was the one that gave no noise or holes in the ROI, such that all pixels in the ROI were properly measured. Disparity varies with the resolution and height of the device, so the disparity was adjusted using trial and error for each height/resolution combination. If pixels in the ROI were not being measured properly, the trial was repeated. In total, 1462 data points were collected with the following features:

- Resolution: Three resolution options were tested for all trials at different heights. The resolutions were high (1280 × 640), medium (640 × 320), and low (424 × 240).
- Distance from the objects: The heights ranged from 130 to 290 mm. Each height at each resolution was recorded three times, and the mean value is used as a feature.
- Light: The data were collected in both indoor and outdoor environments with lights on/off and with sunlight and shade. The order that the data was captured for the light trials was alternated.
- Temperature: The temperature of the RealSense device gradually increases as the device is in use and tends to increase more when the laser projector is enabled. Generally, it takes about 10–15 minutes for the device to reach a steady-state temperature when the laser is not enabled. With the room temperature of 22 °C, the steady-state temperature is in the range of 38–42 °C. Two temperature tests were conducted on the device. In the first experiment, the device was continuously operating in room temperature until it reached a steady state. The device operating temperature and the distance measurements were performed continuously (about every 20 s) as it heated up. In the second experiment, the device was heated up to 60 °C and cooled down to 20 °C by using a heat gun and cooler. The data was also captured continuously every 20 s.

#### 3.1.2. Proposed Error Prediction Models

The performance of prediction models mainly depends on the training data quality, extracted features, and learning algorithms. The prediction model has been formulated as follows.

With *p* extracted features, given a set *W = {w_1_, w*_2_*, …, w_n_}* of captured outputs from the camera and a set *A = {a_1_, a*_2_*, …, a_l_}* of actual errors, the goal is to find the best prediction model C, such that for any *w_k_* that contains a feature set *F_k_ = {f_k,1_, f_k,_*_2_*,…, f_k,p_}*, the predicted error (the difference between the averaged depth of the pixels in the ROI and the true distance of the camera to the ground) $\hat{a}$*_k_ = C(F_k_)* is as identical as possible to the actual error during *w_k_*.

In our experiment, we have initially trained the models with nine features including: (1) resolution (3 levels), (2) indoor/outdoor, (3) actual distance from object (in the range of (130–290 cm)), (4) lighting (4 levels), (5) temperature, (6) projector status (on/off), (7) if heater/cooler is used, (8) the angle of the camera versus level (0°), and (9) the range of the outputs from ROI by the camera defined as maximum distance—minimum distance. Eight different regression techniques were evaluated including Gaussian Process (GP), linear, and quadratic regressions with different parameters; the main objective of implementing different regression models was to review, compare, and evaluate their performance for our error prediction application.

Based on the RealSense documentation, the error increases quadratically as the height increases from 0 to 4 m [37]. In our application, we are interested in low height ranges, as these have the smallest error. Therefore, when the height is from 0 to 1 m, the relationship can be approximated as linear, as it is one small section of the parabola where the slopes are similar. This linearity is also observed when analyzing the true height compared to the measured average height in our dataset. Thus, four linear models were tested including simple linear regression, linear regression with interaction effects, and Robust and Stepwise linear regressions. In order to compare with [37], a quadratic regression was also tested. In addition, we have evaluated three non-linear regression models. The Gaussian Process Regression (GPR) models are non-parametric, Bayesian approaches for a supervised learning problem with a set of random variables where any finite number of them have a joint Gaussian distribution [52,53]. One major limitation of GPR is the computational cost for large datasets [54]. However, studies showed that this method performs well on small datasets, which is the case of our error prediction [55]. GPR models are specified by their mean function *m(x)* and covariance function *k(x,x’)*. The covariance function is usually parameterized by a set of kernel parameters or hyperparameters, *θ* as *k(x,x’|θ)* [56]. In this paper, we used three different types of kernels listed in including Rational Quadratic, Squared Exponential, and 5/2 Matern. We have used 10-fold and holdout cross-validation to prevent possible overfitting and provide better generalization ability.

### 3.2. Crack Detection Algorithm

In this paper, tripping hazards are defined as two surfaces that have a minimum of 6 mm vertical offset from each other. The standard in many municipalities in the USA is to only repair sidewalk cracks that are larger than 6 mm (0.25 in) and 20 mm in Ontario, Canada. These height differences are still potentially a tripping hazard for those with lower Minimum Foot Clearance (MFC) [1,57,58]. The average MFC in an older population is often reported to be less than 20 mm. Therefore, in the proposed algorithm, we have set the threshold value to the US standard of 6 mm. This threshold can be customized based on the required sensitivity and application requirements.

**Figure 2:**
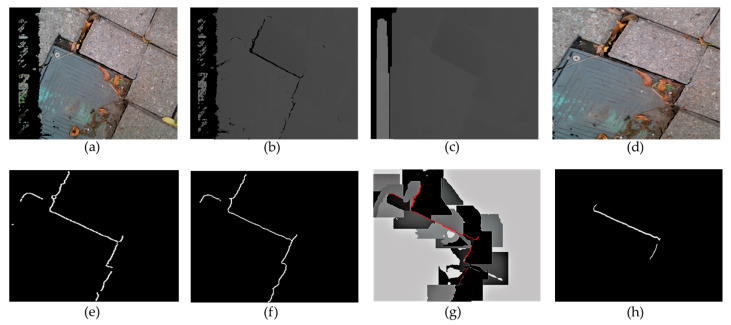
Visualization of each step of the crack detection algorithm showing (**a**) the original tripping hazard in the sidewalk, (**b**) the depth map of the captured frame, (**c**) the filtered depth map, (**d**) the Region of Interest (ROI), (**e**) the edges using Canny Edge Detection Algorithm, (**f**) edges after removing small edges and filling in small gaps between edges, (**g**) the image after segmentation in shades of gray and the true edges shown by the red line, (**h**) the final detection of the tripping hazard.

The captured pictures are first filtered using the Intel RealSense’s built-in filters, including the decimation, spatial, temporal, and hole-filling filters (Figure 2a–c). Then, the user is able to select the desired ROI in a designed Graphical User Interfaces (GUI) in MATLAB. This region can be re-selected at any time or the entire image can be used (Figure 2d). A Canny Edge Detection algorithm is performed to isolate the edges in the images. An example has been shown in Figure 2e. This method first applies a Gaussian filter to reduce the noise of the image. Then, it uses two threshold values to detect the weak and strong edges. The output will include the weak edges only if they are connected to the strong edges. Finally, the edge map is binarized, and edges that are close to each other are merged to obtain Figure 2f.

**Figure 3:**
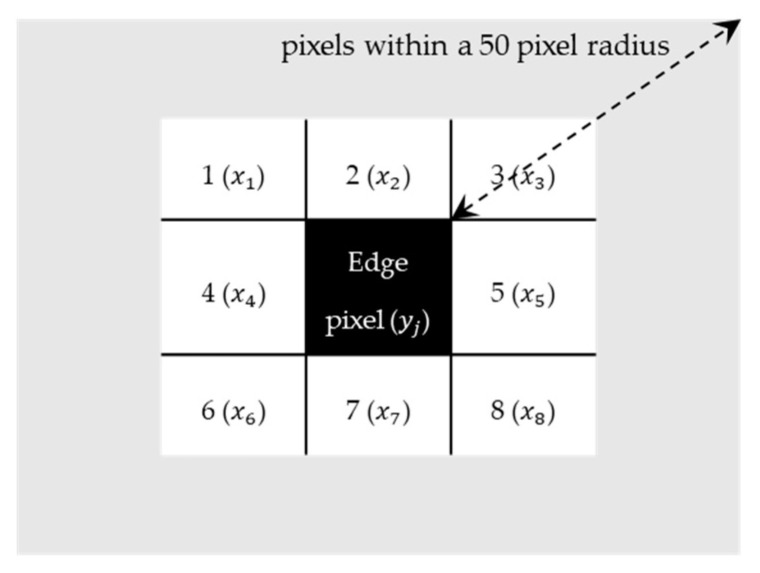
An example of an edge pixel and its eight neighbors in an image.

**Figure 4:**
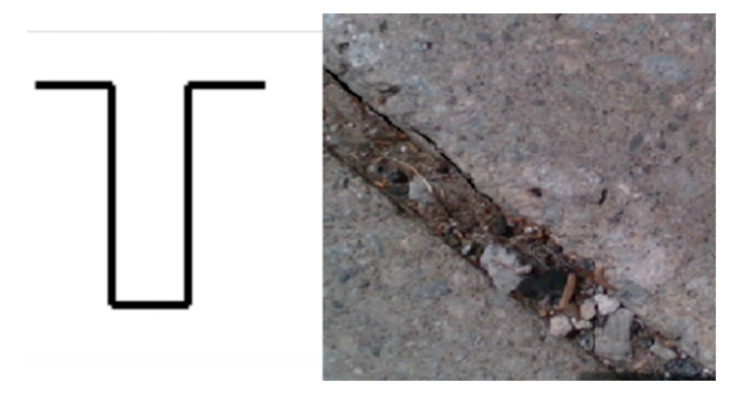
Diagram and an example of a slit.

After these initial steps, the proposed segmentation technique is applied to detect the “true edges”, which are potential edges that can be considered as tripping hazards (Figure 2g). To remove the variations and possible errors of the detected edges, it is essential to perform segmentation around each edge and consider the averaged depth to determine if there was truly an edge. Thus, in this paper, a new segmentation algorithm is proposed that divides the regions surrounding the detected edges (seeds) into segments that include pixels with similar depth. This is done by assigning different numerical labels to the segments. Assume that the pixels of the detected edges are labeled as $\;y_{j}$, and the eight neighboring pixels of the edge are evaluated ($x_{1:8}$), as shown in Figure 3. If the edge pixel and the neighboring pixel have a similar depth (<3 mm), the neighboring pixel is labeled with the same label as the current pixel ($y_{j})$, and the neighbor becomes the next pixel to be analyzed. Pixels with the same labels will be placed in one segment. This step is repeated for all edges’ pixels, until a neighboring pixel $x_{i}$ has a depth difference greater than 3 mm from the current pixel $\;y_{j}$ or until all $x_{i}$ pixels within a 50-pixel radius from $y_{1}$ have been labeled. This cut-off radius has been used to reduce the execution time of the algorithm by only analyzing the regions of interest, which are regions surrounding the detected edges. If the edge pixel and the neighboring pixel have a depth difference greater than 3 mm, the neighboring pixel $x_{i}$ will be relabeled with a different label to show that this pixel belongs to another segment. This step is repeated until all the connected pixels within a 50-pixel radius of the detected edge are labeled. If the difference between the depths of the two pixels is greater than 6 mm, these pixels are labeled as a “true edge”. In Figure 2g, the true edge is shown in red, and the segments are shown in different shades of gray. The different shades are only used to visualize the segments and do not represent any information about the depth values. After the segmentation step, any possible slits are identified by analyzing the distance between each detected crack edge and are removed from the image, as presented in Figure 2h. In this paper, we assume that the slits are not considered as the tripping hazards. An example of a slit is shown in Figure 4. The segmentation process is repeated for each frame captured by the camera while scanning the sidewalk.

## 4. Results and Discussion

### 4.1. Error Prediction Results

In this section, first, we report the outcomes from the proposed error prediction model and then use the estimated error to increase the accuracy of our tripping hazards detection technique. The holdout and 10-fold cross-validation are used to ensure the validity of the error prediction models. For the holdout, we randomly selected 70% of the dataset for training and tested the algorithms on the remaining 30% of the data. For 10-fold cross-validation, the model is trained using nine of the folds as training data, and the obtained model is validated on the remaining part of the data to compute the accuracy. The Mean Absolute Error (MAE) from the 10-fold and holdout cross-validation for each of the eight prediction models are obtained and shown in Table 1. The results showed that the GPR models (non-linear) provide lower MAEs compared to linear and quadratic models for both holdout and 10-fold cross-validation.

Different feature selection (FS) algorithms were used to select features that contribute the most to our prediction model and to reduce any chance of overfitting. We chose to use filter methods, since they do not suffer from the high computational cost associated with repeatedly invoking the learning algorithms, and they can be used with all model types. The chosen filters were mainly from the Feature Selection Library by Roffo [59], including the Correlation-based Feature Selection (CFS), the Local Learning-based Clustering Feature Selection (LLCFS), and the Laplacian Score, which are unsupervised techniques. The Relief-F and Neighboring Component Analysis (NCA) with regularization were also tested, which are included as supervised methods.

In general, CFS can outperform the wrapper on small datasets [60]; therefore, it is an appropriate feature selection for our regression problem. The CFS algorithm attempts to maximize the following objective in its heuristic search strategy [60].

(1) $$M_{s}=\frac{k{\bar{r}}_{cf}}{\sqrt{k+k\left(1\;-\;k\right){\bar{r}}_{ff}}}$$

where *M_s_* is the heuristic merit of feature subset *S* with *k* features, *r**cf* is the average feature-class correlation, and *r**_ff_* is the mean feature–feature inter-correlation. Here, CFS starts from an empty set of features and uses a forward best first search. The search is considered completed once five consecutive fully expanded subsets resulted in no improvement over the current best subset. The LLCFS algorithm uses neighbors to find a good clustering output by a Kernel Machine [61]. A weight is assigned to each feature, which is incorporated into the regularization. Weights are iteratively estimated as the data are clustered [62]. The Laplacian Score has been developed based on the fundamentals of the Laplacian Eigenmap and Locality Preserving Projection [63]. In this method, the locality preserving power is used to determine the importance of the feature, and a nearest neighbor graph is created [63]. The algorithm determines which features follow the graph’s structure to assign it a weight [59]. In this case, the affinity matrix was formed using k-Nearest Neighbor and the Heat Kernel weight mode, which determines how the neighbors are selected and how the graph is modeled [63].

**Figure 5:**
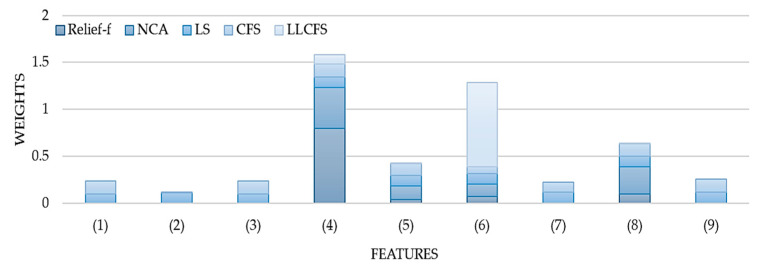
Weights from five feature selection algorithms after normalization.

**Figure 6:**
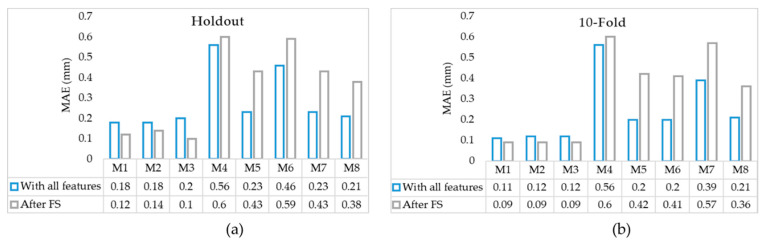
MAEs with and without feature selection for (**a**) holdout with 30% and (**b**) 10-fold cross-validation.

NCA with regularization is a subset of the k-Nearest Neighbor (KNN) algorithm, which searches the neighbors of a trial (i.e., those with similar input data) to determine the expected response [64]. NCA is a non-parametric method where a gradient ascent technique is used with KNN to maximize the accuracy by using a regularization parameter lambda that prevents overfitting [64]. Several lambda values were tested to determine which value resulted in the smallest loss to determine the most accurate predictor weights. The Relief-F algorithm is also a subset of the KNN algorithm, which penalizes predictors that give varying responses to neighbors in the same class and rewards those that do not [65]. It iteratively and randomly chooses a trial, finding the nearest neighboring observations, using intermediate weights to compute the final weight [65]. Figure 5 denotes the normalized weights obtained from all five algorithms. Considering all five methods, the important features are height (4), range (5), temperature (6), and angle error (8). All models were retrained using these four features. The results are shown in Figure 6.

**Figure 7:**
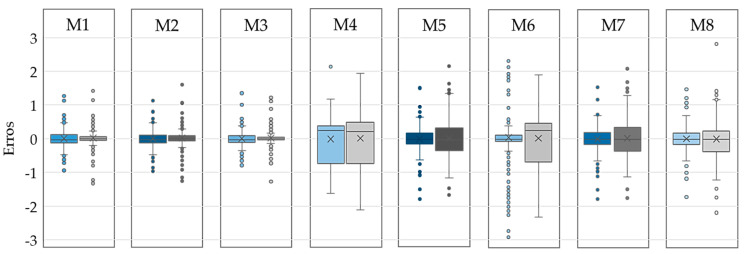
Error bars shown in blue and gray for models without and with feature selection, respectively with holdout validation.

**Figure 8:**
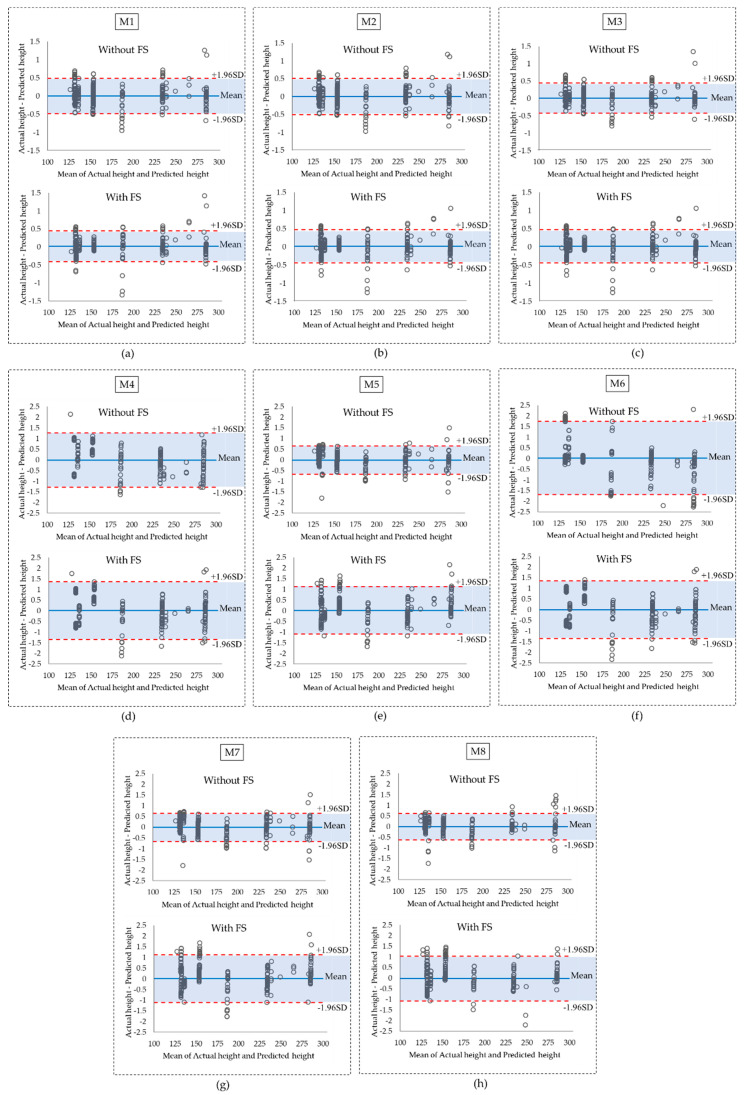
The Bland–Altman plots for error values before and after feature selection for (**a**) M1, (**b**) M2, (**c**) M3, (**d**) M4, (**e**) M5, (**f**) M6, (**g**) M7, and (**h**) M8. The blue shaded areas represent the confidence intervals around the mean.

It was observed that after feature selection, all GPR models (M1, M2, and M3) provide lower MAEs; however, the MAEs for all linear (M4, M5, M6, M7) and quadratic (M8) models increased after feature selection in both holdout and 10-fold cross-validation. This indicated that even though the features that are removed are shown not to be relatively important, they are still contributing to a better error prediction in these models. The GPR models remain the best predictors with MAEs of 0.09 mm for all three models in 10-fold cross-validation. This is also confirmed by the distribution and the standard deviations of the errors in Figure 7. Although the maximum error increases in M1, M2, and M3 after feature selection, the results are more stable compared to the same models trained with all features. This conclusion has also been highlighted in Figure 8a–c in Bland–Altman plots where the confidence interval around the mean values shrinks after feature selection. Furthermore, the linear models M5 and M7 have similar mean errors (MAEs) and upper/lower bounds according to Figure 6a, Figure 7 and Figure 8e,g. However, M5 provides a lower MAE with 10-fold cross-validation with and without feature selections, as seen in Figure 6b. The performance of the quadratic model also decreased after feature selection based on Figure 6, Figure 7, and Figure 8h. Overall, M3 was the best model with the lowest MAE and standard deviation considering both holdout and 10-fold cross-validation.

The RealSense website has shown that height alone has a direct impact on the error [37]. Therefore, we compared the results with simple linear and quadratic regression using height as the only input, as shown in Table 2. This table shows that the other features play a key role in predicting the error in a quadratic model where the error increased from 0.21 to 0.38 mm (≈80%)–0.61 mm (≈190%) with nine, four, and one feature, respectively. However, these addition features do not have a significant effect in predicting the error when using linear regression (≈10% difference when using nine and one features).

### 4.2. Visual Output of the Proposed Crack Detection Algorithm

**Figure 9:**
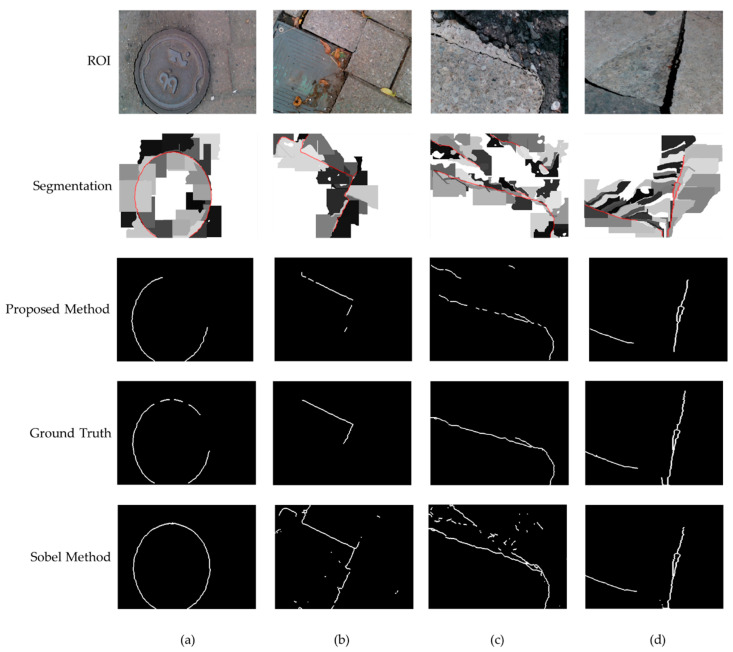
(**a**–**d**) are examples of crack detection algorithm performed on tripping hazards found in the sidewalk where the first row shows the ROI images, the second row shows the images after segmentation with the crack outlined in red, the third row shows the final results of our proposed edge detection algorithm, the fourth row is the ground truth images, and the fifth row shows the output of the conventional Sobel algorithm.

**Figure 10:**
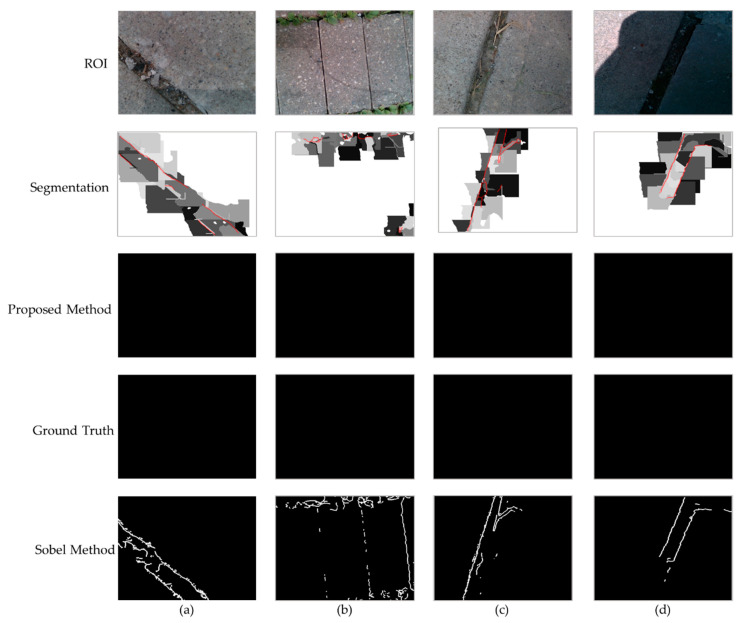
(**a**–**d**) are examples of our crack detection algorithm performed on slits, which are not considered tripping hazards where the first row shows the ROI images, the second row shows the images after segmentation with the crack outlined in red, the third row shows the final result of our edge detection algorithm, the fourth row shows the ground truth images, and the fifth row shows 5. Conclusions and Future Work.

The results of the proposed method are compared with the ground truth obtained from the calibrated images with less than 0.1 mm MAE. First, all pixels that have differences of 6 mm with neighboring pixels are selected; then, a trained researcher manually selected the ground truth based on the visual examination of the color and the depth images. The results are also compared with the conventional Sobel algorithm. This method was selected since both Canny and Sobel are based on the gradients of the images. Sobel uses the convolution of the image and a gradient kernel without any thresholding or hysteresis suppression. Based on the nature of our application, we just need to determine if an image includes any possible tripping hazards, so that the city’s inspectors will be informed of the detection and location of the hazards to investigate the area closely. Therefore, in this case, we need a high precision of detection but not necessarily to detect all the pixels of the hazard. To compare our algorithm with the Sobel method, we determined the precision, recall, and F-measure. For each example, the ROI was selected 10 times and the average precision values obtained 57% and 43% for the proposed and the Sobel algorithm, respectively. Figure 9 and Figure 10 show different examples in one selected ROI.

In our specific application, we are filtering the detected edge pixels based on the definition of the tripping hazards (>6 mm); thus, as expected, the average recall (Sensitivity) was lower in our method compared to Sobel (50% versus 60%). However, the average F-measure of the proposed method was slightly higher (52%) than the conventional one (50%). The main advantage of our proposed method is its low false positive rate, which is highlighted in the examples that contain slits in Figure 10. These examples are excluded from the precision, recall, and F-measure calculations, since they all are perfectly detected with the proposed method with no true positives (edge pixels) or false negatives. As depicted in Figure 10, the conventional Sobel algorithm was over-detecting the slits as cracks. If the slit is wide enough, a foot could fit in the gap, causing a trip. Our algorithm calculates the width of the slit to determine if they are hazardous, so wide slits would still be considered tripping hazards. In our proposed algorithm, a crack is considered a tripping hazard and needs to be repaired if any portion of it is being detected.

The main goal of this study was to provide a new affordable solution for extracting features of walkway tripping hazards. This study focuses on hazards in the form of edges and abrupt discontinuities in sidewalks. However, it is also likely that a trip happens when no edge is present. For example, changes in the slope (gradual humps in the ground) would not be detected by the current algorithm and is one of the limitations of the proposed method. The high accuracy of the Intel RealSense demonstrated in this paper shows that the device also has the potential to detect these types of hazards, which can be the subject of future publications.

The main reason for trip-related falls is inadequate Minimum Foot Clearance (MFC) in the mid-swing phase of the gait cycle when the foot is at maximum speed [66]. The current methods to identify tripping hazards do not account for the variability of the MFC amongst people with different health conditions. Research has found that MFCs for healthy young and older adults are in the range of 12.9 ± 6.2 mm and 11.2 ± 5 mm, respectively [57]. Additionally, evidence shows that MFC can decrease even further, with a many elderly people having an MFC below 6 mm [57]. Therefore, even a small unevenness in the sidewalk can pose a high risk of trips and subsequent falls. Moreover, performing multiple tasks when walking, obstructed visibility, and having fallen in the past can all be factors leading to a significantly lower MFC and subsequently falling [67,68]. Despite the knowledge of the MFC range, cities only repair sidewalk cracks greater than 20 mm in Canada and 6 mm in the USA. This leads to a large unaddressed discrepancy and could result in an increased number of falls [1,58]. Although there is no universally accepted interpretation of the tripping hazards, our tool can provide a vision-based inspection system for tripping hazards assessment. To the best of our knowledge, this is the first study that proposed an automated solution for tripping hazards inspection in sidewalks. The proposed low-cost technique can help cities perform annual inspections.

This paper presents a real-time sidewalk scanner to detect cracks as one type of tripping hazard. The Intel RealSense D415 is used to capture the depth images and generate profiles of the hazards. In the first section of this paper, we proposed a calibration method that uses different features and ML models for predicting the error. The Gaussian Process Regression model yielded the most accurate results to estimate the error. The error is predicted and used to calibrate the initial bias of the camera. In the second part, a novel algorithm was presented to detect cracks in the image by segmenting the calibrated depth map by combining edge detection and region growing. The results were presented using different examples of real-world tripping hazards.

The major limitation of this paper is that only the average depth reading is predicted, not the individual error found in each pixel. It is best used on flat ground, as a calibration technique before the device starts detecting cracks. The average error is subtracted from each individual pixel, which could lead to some inaccuracies, as the actual error of each pixel can vary. Further research must be done to attempt to predict the error in all pixels dynamically and remove them as the device moves. Additionally, the crack detection algorithm will be improved in the future to detect various obstructions, including grass-covered cracks. It can also be expanded to detect slopes and bumps in sidewalks that are not edges but can also contribute to trips and stumbles.

## Figures and Tables

**Table 1:** Mean Absolute Error (MAE) for all models using holdout and 10-Fold validation with all nine features.

Method	Kernel/Specifier	MAE (mm)	
		Holdout	10-Fold
Gaussian Process (M1)	Rational Quadratic	0.18	0.11
Gaussian Process (M2)	Squared Exponential	0.18	0.12
Gaussian Process (M3)	5/2 Matern	0.20	0.12
Linear (M4)	Simple	0.56	0.56
Linear (M5)	with Interactions	0.23	0.20
Linear (M6)	Robust	0.46	0.20
Linear (M7)	Stepwise	0.23	0.39
Quadratic (M8)	NA	0.21	0.21

**Table 2:** MAE for simple linear and quadratic models with a different number of features.

Method	MAE (mm)					
	9 Features		4 Features		1 Feature	
	Holdout	10-fold	Holdout	10-fold	Holdout	10-fold
**Linear (M5)**	0.56	0.56	0.60	0.60	0.63	0.62
**Quadratic (M9)**	0.21	0.21	0.38	0.36	0.61	0.60

## References

[1] Minimum Maintenance Standards (2014). Minimum Maintenance Standards for Municipal Highways Policy.

[2] International Code Council (ICC) (2014). Chapter 10 Means of Egress. International Building Code.

[3] Fekr A.R., Evans G., Fernie G., Bagnara S., Tartaglia R., Albolino S., Alexander T., Fujita Y. (2019). Walkway Safety Evaluation and Hazards Investigation for Trips and Stumbles Prevention. Proceedings of the 20th Congress of the International Ergonomics Association (IEA 2018).

[4] Green J. (2015). 89-Year-Old Wins $192K in Court after Tripping on Cracked Hamilton Sidewalk CBC News. https://www.cbc.ca/news/canada/hamilton/headlines/89-year-old-wins-192k-settlement-after-tripping-on-cracked-hamilton-sidewalk-1.3108350.

[5] Ai C., James Tsai Y. (2016). An Automated Sidewalk Assessment Method for the Americans with Disabilities Act Compliance Using 3-D Mobile LiDAR. Transp. Res. Rec. J. Transp. Res. Board.

[6] McMahon S., Sünderhauf N., Upcroft B., Milford M. (2017). Multimodal Trip Hazard Affordance Detection on Construction Sites. IEEE Robot. Autom. Lett..

[7] Bitelli G., Simone A., Girardi F., Lantieri C. (2012). Laser Scanning on Road Pavements: A New Approach for Characterizing Surface Texture. Sensors.

[8] Annovi A., Fechney R., Yenkanchi S., Lowe D., Shah H., Sivakumar P.K., Singh I.P., Galchinsky M. (2019). High Speed Stereoscopic Pavement Surface Scanning System and Method. U.S. Patent.

[9] Zou Q., Cao Y., Li Q., Mao Q., Wang S. (2012). CrackTree: Automatic crack detection from pavement images. Pattern Recognit. Lett..

[10] Tanaka N., Uematsu K. A Crack Detection Method in Road Surface Images Using Morphology. Proceedings of the MVA 1998.

[11] Xu B., Huang Y. (2010). Automated Surface Distress Measurement System. U.S. Patent.

[12] Zhang D., Li Q., Chen Y., Cao M., He L., Zhang B. (2017). An efficient and reliable coarse-to-fine approach for asphalt pavement crack detection. Image Vis. Comput..

[13] Oliveira H., Correia P.L. (2013). Automatic Road Crack Detection and Characterization. IEEE Trans. Intell. Transp. Syst..

[14] Ferguson R.A., Pratt D.N., Turtle P.R., MacIntyre I.B., Moore D.P., Kearney P.D., Best M.J., Gardner J.L., Berman M., Buckley M.J. (2003). Road Pavement Deterioration Inspection System. U.S. Patent.

[15] Jia Y., Tang L., Xu B., Zhang S. (2019). Crack Detection in Concrete Parts Using Vibrothermography. J. Nondestruct. Eval..

[16] Fujita Y., Hamamoto Y. (2011). A robust automatic crack detection method from noisy concrete surfaces. Mach. Vis. Appl..

[17] Yamaguchi T., Hashimoto S. (2010). Fast crack detection method for large-size concrete surface images using percolation-based image processing. Mach. Vis. Appl..

[18] Efficient Pavement Crack Detection and Classification SpringerLink. https://link.springer.com/article/10.1186/s13640-017-0187-0.

[19] Qu Z., Ju F.-R., Guo Y., Bai L., Chen K. (2018). Concrete surface crack detection with the improved pre-extraction and the second percolation processing methods. PLoS ONE.

[20] Zhang L., Yang F., Zhang Y.D., Zhu Y.J. Road crack detection using deep convolutional neural network. Proceedings of the 2016 IEEE International Conference on Image Processing (ICIP).

[21] Bertuletti S., Cereatti A., Comotti D., Caldara M., Della Croce U. (2017). Static and Dynamic Accuracy of an Innovative Miniaturized Wearable Platform for Short Range Distance Measurements for Human Movement Applications. Sensors.

[22] Glenn N.F., Streutker D.J., Chadwick G.D., Thackray G.D., Dorsch S.J. (2006). Analysis of LiDAR-derived topographic information for characterizing and differentiating landslide morphology and activity. Geomorphology.

[23] Lohani B., Mohan K. (2007). Airborne Altimetric LiDAR: Principle, Data Collection, Processing and Applications.

[24] Light Detection and Ranging (LiDAR) Presented at Portland State University. http://web.pdx.edu/~jduh/courses/geog493f12/Week04.pdf.

[25] Gavilán M., Balcones D., Marcos O., Llorca D.F., Sotelo M.A., Parra I., Ocaña M., Aliseda P., Yarza P., Amírola A. (2011). Adaptive road crack detection system by pavement classification. Sensors.

[26] Li X.-Q., Wang Z., Fu L.-H. (2016). A Laser-Based Measuring System for Online Quality Control of Car Engine Block. Sensors.

[27] Laser Triangulation Sensors M.T.I Instruments. https://www.mtiinstruments.com/technology-principles/laser-triangulation-sensors/.

[28] Alhwarin F., Ferrein A., Scholl I., Pham D.-N., Park S.-B. (2014). IR Stereo Kinect: Improving Depth Images by Combining Structured Light with IR Stereo. Proceedings of the PRICAI 2014: Trends in Artificial Intelligence.

[29] Banks M.S., Read J.C.A., Allison R.S., Watt S.J. (2012). Stereoscopy and the Human Visual System. SMPTE Motion Imaging J..

[30] Giancola S., Valenti M., Sala R. (2018). A Survey on 3D Cameras: Metrological Comparison of Time-of-Flight, Structured-Light and Active Stereoscopy Technologies.

[31] (2018). Choosing an Intel® RealSenseTM Depth Camera. https://www.intelrealsense.com/compare/.

[32] Webster J. (2016). Structured Light Techniques and Applications.

[33] Vit A., Shani G. (2018). Comparing RGB-D Sensors for Close Range Outdoor Agricultural Phenotyping. Sensors.

[34] Carfagni M., Furferi R., Governi L., Santarelli C., Servi M., Uccheddu F., Volpe Y. (2019). Metrological and Critical Characterization of the Intel D415 Stereo Depth Camera. Sensors.

[35] Intel® RealSenseTM Depth Camera D415. https://store.intelrealsense.com/buy-intel-realsense-depth-camera-d415.html.

[36] (2019). Intel RealSense D400 Series Calibration Tools-User Guide; Intel RealSense. https://dev.intelrealsense.com/docs/intel-realsensetm-d400-series-calibration-tools-user-guide.

[37] Grunnet-Jepsen A., Sweetser J.N., Woodfill J. (2018). Best-Known-Methods for Tuning Intel^®^ RealSense^TM^ D400 Depth Cameras for Best Performance.

[38] Zhang Z. (2000). A flexible new technique for camera calibration. IEEE Trans. Pattern Anal. Mach. Intell..

[39] Fuersattel P., Plank C., Maier A., Riess C. (2017). Accurate laser scanner to camera calibration with application to range sensor evaluation. IPSJ Trans. Comput. Vis. Appl..

[40] Cabrera E.V., Ortiz L.E., da Silva B.M.F., Clua E.W.G., Gonçalves L.M.G. (2018). A versatile method for depth data error estimation in RGB-D sensors. Sensors.

[41] Chen G., Cui G., Jin Z., Wu F., Chen X. (2019). Accurate Intrinsic and Extrinsic Calibration of RGB-D Cameras with GP-Based Depth Correction. IEEE Sens. J..

[42] Yamazoe H., Habe H., Mitsugami I., Yagi Y. (2018). Depth error correction for projector-camera based consumer depth cameras. Comput. Vis. Media.

[43] Adams R., Bischof L. (1994). Seeded region growing. IEEE Trans. Pattern Anal. Mach. Intell..

[44] Isa N.A.M. (2005). Automated Edge Detection Technique for Pap Smear Images Using Moving K-Means Clustering and Modified Seed Based Region Growing Algorithm. Int. J. Comput. Internet Manag..

[45] Fan J., Yau D.K.Y., Elmagarmid A.K., Aref W.G. (2001). Automatic image segmentation by integrating color-edge extraction and seeded region growing. IEEE Trans. Image Process..

[46] Wang N., Yang J. (2011). Color image segmentation by edge linking and region grouping. J. Shanghai Jiaotong Univ. (Sci.).

[47] Pavlidis T., Liow Y.-T. (1990). Integrating region growing and edge detection. IEEE Trans. Pattern Anal. Mach. Intell..

[48] Luo Y., Liu L., Huang Q., Li X. (2017). A Novel Segmentation Approach Combining Region- and Edge-Based Information for Ultrasound Images. BioMed Res. Int..

[49] Chen H., Ding H., He X., Zhuang H. Color image segmentation based on seeded region growing with Canny edge detection. Proceedings of the 2014 12th International Conference on Signal Processing (ICSP).

[50] Yi-Wei Y., Wang J.-H. Image segmentation based on region growing and edge detection. Proceedings of the IEEE SMC’99 Conference Proceedings. 1999 IEEE International Conference on Systems, Man, and Cybernetics (Cat. No.99CH37028).

[51] Al-Hujazi E., Sood A. (1990). Range image segmentation combining edge-detection and region-growing techniques with applications sto robot bin-picking using vacuum gripper. IEEE Trans. Syst. Man Cybern..

[52] Schulz E., Speekenbrink M., Krause A. (2018). A tutorial on Gaussian Process Regression: Modelling, Exploring, and Exploiting Functions. J. Math. Psychol..

[53] Zhang N., Xiong J., Zhong J., Leatham K. Gaussian Process Regression Method for Classification for High-Dimensional Data with Limited Samples. Proceedings of the 2018 Eighth International Conference on Information Science and Technology (ICIST).

[54] Idé T., Kato S. Travel-Time Prediction using Gaussian Process Regression: A Trajectory-Based Approach. Proceedings of the 2009 SIAM International Conference on Data Mining; Society for Industrial and Applied Mathematics.

[55] Wang J.M., Fleet D.J., Hertzmann A. (2008). Gaussian Process Dynamical Models for Human Motion. IEEE Trans. Pattern Anal. Mach. Intell..

[56] MacKay D.J. (1992). Bayesian interpolation. Neural Comput..

[57] Begg R., Best R., Dell’Oro L., Taylor S. (2007). Minimum foot clearance during walking: Strategies for the minimisation of trip-related falls. Gait Posture.

[58] Boodlal L. (2004). Accessible Sidewalks and Street Crossings: An Informational Guide.

[59] Roffo G. Feature Selection Library (MATLAB Toolbox). MATLAB Central File Exchange. https://www.mathworks.com/matlabcentral/fileexchange/56937-feature-selection-library.

[60] Soliman O.S., Rassem A., Sombattheera C., Loi N.K., Wankar R., Quan T. (2012). Correlation Based Feature Selection Using Quantum Bio Inspired Estimation of Distribution Algorithm. Multi-disciplinary Trends in Artificial Intelligence.

[61] Wu M., Schölkopf B., Schölkopf B., Platt J.C., Hoffman T. (2007). A Local Learning Approach for Clustering. Advances in Neural Information Processing Systems 19.

[62] Zeng H., Cheung Y., Theeramunkong T., Kijsirikul B., Cercone N., Ho T.-B. (2009). Feature Selection for Local Learning Based Clustering. Proceedings of the Advances in Knowledge Discovery and Data Mining.

[63] He X., Cai D., Niyogi P., Weiss Y., Schölkopf B., Platt J.C. (2006). Laplacian Score for Feature Selection. Advances in Neural Information Processing Systems 18.

[64] Yang W., Wang K., Zuo W. (2012). Neighborhood Component Feature Selection for High-Dimensional Data. JCP.

[65] Robnik-Sikonja M., Kononenko I. (1997). An adaptation of Relief for Attribute Estimation in Regression. Proceedings of the Machine Learning: Proceedings of the Fourteenth International Conference (ICML’97).

[66] Winter D.A. (1992). Foot trajectory in human gait: A precise and multifactorial motor control task. Phys. Ther..

[67] Chiba H., Ebihara S., Tomita N., Sasaki H., Butler J.P. (2005). Differential gait kinematics between fallers and non-fallers in community-dwelling elderly people. Geriatr. Gerontol. Int..

[68] Schulz B.W., Lloyd J.D., Lee W.E. (2010). The Effects of Everyday Concurrent Tasks on Overground Minimum Toe Clearance and Gait Parameters. Gait Posture.

